# The Skin–Brain Axis: From UV and Pigmentation to Behaviour Modulation

**DOI:** 10.3390/ijms25116199

**Published:** 2024-06-04

**Authors:** Anna A. Ascsillán, Lajos V. Kemény

**Affiliations:** 1 Department of Dermatology, Venereology and Dermatooncology, Faculty of Medicine, Semmelweis University, 1085 Budapest, Hungary; 2 HCEMM-SU Translational Dermatology Research Group, Semmelweis University, 1094 Budapest, Hungary; 3 Department of Physiology, Faculty of Medicine, Semmelweis University, 1094 Budapest, Hungary; 4 Division of Infection and Immunity, University College London, London WC1E 6BT, UK

**Keywords:** skin, central nervous system, skin–brain axis, melanocyte, keratinocyte, melanogenesis, pigmentation, UVR, HPA axis, ACTH, MSH, cortisol, POMC, eumelanin, pheomelanin, neuromelanin, MC1R, MITF, Parkinson’s disease, tyrosinase, vitamin D, redhead, melanoma, nociception, opioid signalling, Addison’s disease

## Abstract

The skin–brain axis has been suggested to play a role in several pathophysiological conditions, including opioid addiction, Parkinson’s disease and many others. Recent evidence suggests that pathways regulating skin pigmentation may directly and indirectly regulate behaviour. Conversely, CNS-driven neural and hormonal responses have been demonstrated to regulate pigmentation, e.g., under stress. Additionally, due to the shared neuroectodermal origins of the melanocytes and neurons in the CNS, certain CNS diseases may be linked to pigmentation-related changes due to common regulators, e.g., MC1R variations. Furthermore, the HPA analogue of the skin connects skin pigmentation to the endocrine system, thereby allowing the skin to index possible hormonal abnormalities visibly. In this review, insight is provided into skin pigment production and neuromelanin synthesis in the brain and recent findings are summarised on how signalling pathways in the skin, with a particular focus on pigmentation, are interconnected with the central nervous system. Thus, this review may supply a better understanding of the mechanism of several skin–brain associations in health and disease.

## 1. Introduction

The skin, acting as a barrier, protects the organs from external physical and chemical stressors and pathogens, alongside managing water and heat retention as part of the osmoregulatory and thermoregulatory systems. In cooperation with the nervous system, the skin also intercepts environmental stimuli and transmits them further [1]. One feedback loop and signalling system in which the skin actively partakes is the hypothalamic–pituitary–adrenal axis (HPA axis), which involves the central nervous and endocrine systems to adjust to stress. The skin communicates stress towards the central nervous system (CNS) and reacts according to its hormonal response [2]. Although the skin’s involvement in regulating the CNS has been widely covered, this review discusses recent discoveries on the role of skin melanocytes and pigmentation in regulating central responses and their role in health and disease.

## 2. Pigmentation in Melanocytes

### 2.1. Extrinsic Regulation of Pigmentation in Melanocytes

Human skin is the primary barrier protecting the internal organs from external stressors, thus enabling timely responses to toxins and allergens, ultraviolet radiation (UVR), among other forms of radiation, temperature, physical trauma, and pathogens, and communicates signals to the immune, nervous and endocrine systems [3,4]. One of the skin’s main functions is pigment production, which is crucial in maintaining local and global homeostasis. Hundreds of genes regulating pigment production have been under evolutionary selection even in recent human evolution, balancing the need for vitamin D and protection against genotoxic UV radiation [5].

Human melanocytes, derived from neural crest tissue [6], are found primarily in the epidermis. These are the primary pigment-producing cells. However, it should be noted that other cell types have also been described to produce pigment (melanocytes in hair follicles, but also of the mucosa, cochlea, iris, mesencephalon and others) [7]. The brown pigment eumelanin and the red pigment pheomelanin are produced within organelles called melanosomes, which are distributed in the epidermal unit to form microparasols [8]. The pigmentary function of these melanosomes can be further divided into four stages based on their maturation [7,9]. Melanogenesis requires its own set of enzymes partaking in pigment production, along with proteins construing the structure of melanosomes and regulating their distribution [10].

Melanogenesis in melanocytes is regulated by multiple extrinsic (mainly UV) and intrinsic (primarily hormonal) factors. Other than a genetic predisposition to a specific hair, eye and skin colour, which is largely defined as the ratio of black–brown eumelanin to red–yellow pheomelanin that is produced by melanocytes and accumulates in keratinocytes, UVR exposure is the leading extrinsic determinant of skin pigmentation [4]. However, additional DNA-damaging stimuli can trigger the same pathway (i.e., ionising radiation) [11]. UVR induces DNA damage in keratinocytes, leading to p53 activation and proopiomelanocortin (POMC) transcription [12]. POMC is then cleaved to α-MSH, the ligand for the melanocortin-1 receptor (MC1R) on melanocytes. MC1R polymorphisms, due to loss-of-function variations in MC1R, are responsible for the incapability of eumelanin pigment production, leading to sole pheomelanin synthesis. However, when functional MC1R is activated on melanocytes, melanocytes will produce pigment to transport back to keratinocytes to prevent further DNA damage. It is also important to mention that along with MSH production, β-endorphin is also cleaved from POMC, contributing to various behaviour changes described below [13].

### 2.2. Intrinsic Regulation of Pigmentation in Melanocytes

Besides stimulated melanogenesis, intrinsic control of melanocytes has also been established. Multiple paracrine factors and neuroendocrine hormones regulate the signalling pathways and compounds related to the biosynthesis of the two pigments [14], as shown in Table 1.

The dark pigment eumelanin is considered to possess antioxidant activity and also to protect from extensive UVR, in contrast to pheomelanin, a lighter yellow-red pigment with pro-oxidant features and inability to shield from UVR [15]. Pheomelanin even contributes to UV-independent melanoma risk independently, as pheomelanin-producing melanocytes exhibit increased reactive oxygen species (ROS)-induced DNA damage [16]. Both pigments are products of melanogenesis, and their ratio is primarily determined by tyrosinase activity and cysteine availability within melanocytes [17]. However, mainly eumelanin is synthesised in the presence of cysteine and sufficient MC1R activity [18]. α-MSH, a paracrine factor secreted by keratinocytes as a result of UVR exposure, binds to MC1R as an agonist and activates adenylyl cyclase [19], ultimately generating a cyclic AMP (cAMP) signalling cascade for the transcription of the MITF and its downstream transcriptional targets, which are enzymes involved in eumelanin synthesis, namely tyrosinase, tyrosinase-related protein 1, and tyrosinase-related protein 2 (Tyr, TRP1, TRP2) [20].

**Figure 1:**
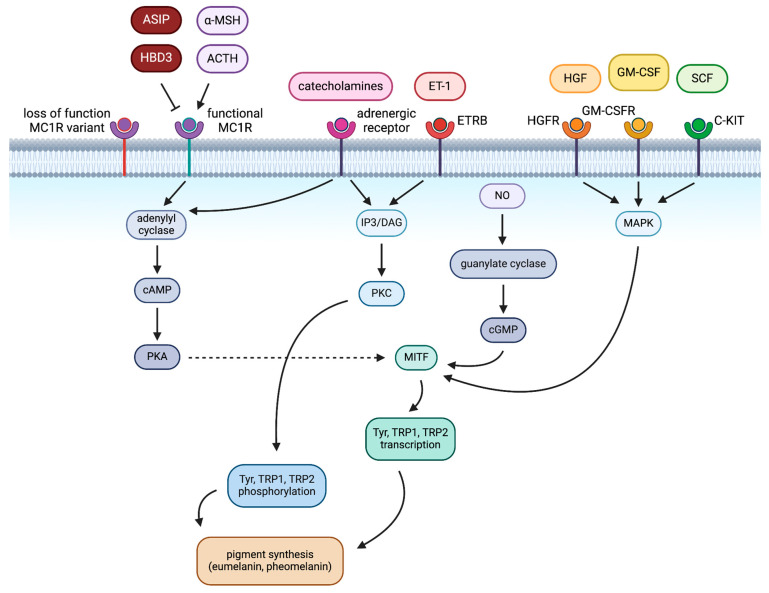
Regulation of pigmentation in melanocytes. Loss-of-function melanocortin-1 receptor (MC1R) is unresponsive to MC1R ligands. Functional MC1R, upon binding adrenocorticotropic hormone (ACTH) or alpha-melanocyte signalling hormone (α-MSH), increases microphthalmia-releasing transcription factor (MITF) expression through protein kinase A (PKA) production. Adrenergic receptors and endothelin receptor type B (ETRB) employ protein kinase B (PKB) to increase tyrosinase (Tyr), tyrosinase-related protein 1 (TRP1) and tyrosinase-related protein 2 (TRP2) phosphorylation, leading to melanogenesis. Nitric oxide (NO) elevates cyclic guanosine monophosphate (cGMP) levels to promote MITF; meanwhile, the hepatocyte growth factor receptor (HGFR), the granulocyte-macrophage colony-stimulating factor receptor (GM-CSFR) and the stem cell factor receptor (C-KIT) do so through mitogen-activated protein kinase (MAPK) signalling. Essentially, these pathways all converge to regulate pigment synthesis. Other abbreviations: agouti-signalling protein (ASIP), human β-defensin 3 (HBD3), endothelin-1 (ET-1), hepatocyte growth factor (HGF), granulocyte-macrophage colony-stimulating factor (GM-CSF), stem cell factor (SCF).

Activating adenylyl cyclase increases intracellular cAMP, activating an effector, cAMP-dependent protein kinase A (PKA). PKA partakes in phosphorylating metabolic enzymes and regulating signalling pathways [21]. Upon the increase in intracellular cAMP, cAMP binds PKA, leading to the PKA-CREB pathway: Adenylyl cyclase activates CREB, thus increasing MITF and promoting melanogenesis [22]. Mitogen-activated protein kinase (MAPK), inositol triphosphate/diacylglycerol (IP3/DAG), Wingless-type protein (WNT), and protein kinase C (PKC) signalling pathways also contribute to the regulation of melanogenesis [23]. For example, the IP3/DAG pathway is employed to increase intracellular PKC-β levels and activate Tyr via the α1 adrenoreceptor [22]. Catecholamines promote melanogenesis through the cAMP/PKA and PKC-β pathways by α1 and β2 adrenoreceptors [22]. (These pathways are schematically summarised in Figure 1).

Agouti signalling protein (ASIP), on the other hand, an antagonist of MC1R (Figure 1), promotes pheomelanin synthesis by blocking the binding of α-MSH and simultaneously affecting MITF downstream expression, necessary for promoter activation of Tyr, TRP1 and TRP2 genes [24]. MC1R receptor deactivation enables the pheomelanin synthesis pathway by reducing the transcription of the genes Tyr and TRP1, among the others mentioned [25].

In melanocytes, when melanosomes reach their final mature form and get distributed to keratinocytes, autophagy within these cells regulates skin colour significantly through controlling the loss of melanosomes [26]. The rate at which this occurs differs in each ethnic background, one mechanism implied previously, through varying protease-activated receptor-2 regulation [27]. Autophagy-related protein (ATG7) and 1 light chain 3 (LC3)-like protein are also autophagy effector proteins. ATG7 knockdown decreases microphthalmia-related transcription factor (MITF) expression, whereas its overexpression causes the opposite [26]. Furthermore, LC3 knockdown suppresses melanocyte-stimulating hormone (α-MSH)-mediated melanogenesis by reducing MITF expression and cAMP-response element binding protein (CREB) phosphorylation [26]. In vitiligo, the loss of melanocytes might result from dysregulated autophagy-induced oxidative stress [28,29].

Melanogenesis occurs in melanocytes, yet as part of the epidermal melanin unit comprising mainly keratinocytes and melanocytes, paracrine factors secreted by keratinocytes can regulate pigment synthesis [30]. Some melanogenesis-promoting factors are interleukin 18 and 33 (IL-18, IL-33), granulocyte-macrophage colony-stimulating factor (GM-CSF, which acts via the MAPK pathway, shown in Figure 1), prostaglandin E2 (PGE2) and prostaglandin F2 (PGF2), while tumour necrosis factor (TNF), interleukin 1 (IL-1, both IL-1α and IL-1β) and interleukin 6 (IL-6) inhibit the process [22]. Other skin cell types, such as fibroblasts, interact with melanocytes via paracrine secretion [31]. The relevance of these paracrine factors is highlighted by the maintenance of melanocyte homeostasis and UV mutagenesis prevention, which are performed mediately to preserve DNA [32].

## 3. Pigmentation in the CNS—Role of Neuromelanin

### 3.1. Neuromelanin in Dopaminergic Neurons

Besides eumelanin and pheomelanin synthesis in the skin, some neurons produce similarly structured neuromelanin pigment. Neuromelanin, consisting of granules akin to those in human hair and eye melanosomes, is a type of melanin formed as a by-product of dopamine auto-oxidation in catecholaminergic neurons [33,34]. In contrast to other melanins, neuromelanin formation is spontaneous, not enzymatically regulated, occurring upon catecholamine release, from dopamine and other catecholamines [35]. The need to create neuromelanin arises because catecholamines pose an oxidative risk for the dopaminergic or adrenergic neurons in the form of quinones [36]. When sequestered into synaptic vesicles, dopamine is stable and not at risk of auto-oxidization [37]. Yet when cytosolic concentrations of dopamine rise as a result of overloading the firing neuron, antioxidant defences, including cysteine and glutathione, are activated before all the released dopamine may be sequestered into vesicles [38]. As such, the fact that neuromelanin’s pheomelanin core contains cysteine becomes evident [39], while its outer eumelanin surface layer contains none: its synthesis occurs later when cysteine levels are already depleted [33,40].

The accumulative nature of neuromelanin synthesis is shown in the black pigmentation of the ageing brain, specifically in the substantia nigra pars compacta and locus coeruleus [33]. In the catecholaminergic neurons of these regions and the brain itself, neuromelanin provides neuronal protection from oxidative stress by sequestering toxic by-products of the neurons’ metabolism [41]. Neuromelanin binds organic amines and metal ions that catalyse the formation of ROS, most prominently iron, along with Zn, Se, Cr, Sr, Sb, Co, Ni, Ce, Hg, Au, Ag, Ta, and Sc [42,43]. Its capacity to do so stems from the difference in its biosynthesis from skin melanin. In contrast to skin eumelanin synthesis, which is enzymatically mediated and produces a multilayer stacked structure on the surface of the melanin’s pheomelanin core, neuromelanin synthesis involves spontaneous eumelanin formation, which concludes in chains of melanic oligomers, contained in lipid- and protein-rich double membrane organelles later on [25,44].

### 3.2. Neuromelanin in Parkinson’s Disease

On the other hand, the neuromelanin that has accumulated with age, accompanied by Parkinson’s Disease (PD)-related mutations, may be a causative factor in ageing-related neurodegeneration, especially when taking into account that, in most cases, no central genetic basis for PD has been identified thus far [25,33]. The build-up of undegraded autophagic structures obstructs cytosolic interaction of cellular components and limits new vesicular storage capacity, also interfering with lysosomal proteases, intracellular trafficking and endo- and exocytic functions [45]. Autophagy dysregulation affects neuronal loss similarly to the process described before in the case of vitiligo: neuromelanin carries undegraded cellular components, transition metals, lipids, etc., to a certain limit [46]. Lipid peroxides and iron dyshomeostasis lead to oxidative stress, and should autophagy dysfunction arise, ROS may induce neuron degeneration and the release of neuromelanin-sequestered toxins, leading to exacerbated immune response [47]. In PD patients, the loss of nigral neurons in the substantia nigra pars compacta (SNpc) is visible to the naked eye, as described by Jean Lhermitte, with the black pigmentation missing in part [48,49]. The degeneration of pigmented dopaminergic neurons leads to the characteristic motor function symptoms in PD patients [50]. The similar neuromelanin-induced loss of noradrenergic neurons in the locus coeruleus and the dorsal motor nucleus of the vagus produce its non-motor symptoms [51]. Meanwhile, neuronal loss in the non-pigmented brain was not found to be consistent with PD [49]. Neuromelanin degenerates dopaminergic neurons by shortening and reducing the number of dendrites and limiting dopamine intake (dysfunctional dendritic mitochondria) [52,53], and by releasing cytochrome C, it also induces mitochondria-mediated apoptosis [54]. Extracellular neuromelanin induces microglial activation [55], an auto-immune response towards dopaminergic neurons, by producing pro-inflammatory factors, nitric oxide, superoxide and hydrogen peroxide [42].

With the gravity of pigment-related pathways in neurodegeneration and PD established, the link between human melanogenesis and PD becomes relevant. In general, PD patients show a lower risk of developing any type of cancer except for one: Cutaneous Malignant Melanoma (CMM) [56]. This link found by epidemiological studies also extends to skin and hair pigmentation at a younger age [57]. As with CMM, people of darker (brown/black) skin and hair show lower PD incidence rates compared to people on the lighter end of the spectrum (blonde/red hair and fair/freckled skin) [58]. Numerous genes and their variants known to determine pigmentation have been linked to PD, such as GCH1, HERC2, LRRK2, OCA2, PRKN, SNCA, TPCN2, TRPM7, TYRP1 and VPS35 [25]. Still, the most promising linking genes thus far are those encoding MC1R and Tyr, elaborated upon previously, along with GPNMB and GCH1 through α-synuclein interactions, among others [59,60]. The identified PD genes PRKN and LRRK2 also partake in pigmentation processes [61].

### 3.3. MC1R in Relation to Parkinson’s Disease

MC1R-related connections made between PD and the pigmentary system are further established as per a recent study, where it was found that peripheral MC1R activation mediated neuroprotective effects in PD models [62]. [Nle^4^, DPhe^7^]-α-MSH (NDP-MSH, α-MSH analogue) is an agonist of MC1R and was found to exert its neuroprotective effects on immune cells to restore the blood–brain barrier (BBB)—disrupted by oxidative stress and neuroinflammation—[63] along with reversing nigral T-cell infiltration (precedes nigral synucleinopathy) [64] and ameliorating oxidative stress and apoptosis of affected neurons [65]. When NDP-MSH is administered peripherally, it does not affect the CNS as it cannot permeate the blood–brain barrier (BBB). Instead, upon binding to peripheral MC1R, it protects dopaminergic neurons via the CREB/Nr4a1/NF-κB pathway (abbreviations: cAMP response element-binding protein/nuclear receptor subfamily 4 group A member 1/nuclear factor kappa B), mitigating PD neuroinflammation, meaning less circulating TNF-α, IL-1β, monocytes and cytotoxic CD8+ T cells [62,66].

A prominent frontier in linking PD, melanogenesis and melanoma is α-synuclein [67]. Inversely correlating with melanin content when expressed in skin, α-synuclein is also found in neuromelanin organelles and shows elevated levels in CMM [68]. α-synuclein controls the aggregation properties of the protein PMEL17 (melanocytic-specific glycoprotein, a functional amyloid protein), which serves as scaffolding for melanin synthesis in human and skin melanoma cell lines [68]; in dopaminergic neurons, α-synuclein overexpression has neurotoxic effects [69] and causes neuromelanin accumulation [70]. CMM-linked α-synuclein depletion coincides with α-synucleinopathy neuronal degeneration in the SNpc in PD; α-synuclein and Tyr interactions also suggest similar interplays [25]. Neuronal MC1R, genetically identical to melanogenesis-regulating cutaneous MC1R, was studied concerning the neurotoxicity of α-synuclein, inspired by the epidemiological associations between MC1R, melanogenesis and melanoma, with the latter also associated with PD (along with red hair and MC1R variants) [58]. α-synuclein accumulation and aggregation, due to mutations in the SNCA gene encoding for α-synuclein, induce proteinopathy, neuroinflammation and oxidative stress, leading to the degeneration and loss of dopaminergic neurons as part of PD pathogenesis [71]. Proteostasis, the redox system and inflammatory processes in PD can all be regulated via the nuclear factor erythroid 2-related factor 2 (Nrf2) [72]. According to a recent study, Nrf2 activation in the substantia nigra is mediated by neuronal MC1R [73]. However, it should be noted that polymorphisms of MC1R are associated with increased melanoma risk not only due to decreased pigment shielding but also due to additional factors, e.g., decreased DNA repair [74,75].

## 4. Skin–Brain Axis Basics—Involvement of the Skin in the HPA Axis

The skin–brain axis consists of bidirectional communication through which CNS responses to psychological stress result in altered skin function; meanwhile, the skin itself can also signal stress to the brain [76]. The interplay occurs on multiple levels, from innervation to the HPA and sympathetic–adrenal medullary (SAM) axes [77]. The skin functions as a peripheral HPA axis, and serotonin, a neurotransmitter in the CNS, has been observed to take effect on the skin [78,79]. Through innervation, the skin is a major interceptor of external stress, transmitting thermal, noxious and mechanical stimuli towards the CNS via thermoreceptors, nociceptors and mechanoreceptors through the spinal cord; cutaneous sensory fibres intercept and communicate changes in temperature, pH and inflammatory mediators [80]. The CNS nerves that transmit skin input signals often terminate near receptors in the brain, enabling direct responses that mediate stress responses in the skin [81]. The stress mediators produced by the CNS to elicit skin response do not only exert their effects but are also produced locally in the skin, triggering immune, inflammatory and pigmentary responses [82]. Other examples of hormones and neuromodulators produced by the central endocrine system which regulate functions of the skin are gluco- and mineralocorticoids, sex hormones, thyroid hormones, growth hormone, prolactin, and adrenocorticotropic hormone (ACTH) and various neuropeptides, -transmitters and biogenic amines [3]. This review focuses on the implications of the skin–brain axis concerning the pigmentary system.

### 4.1. Central HPA Axis

**Figure 2:**
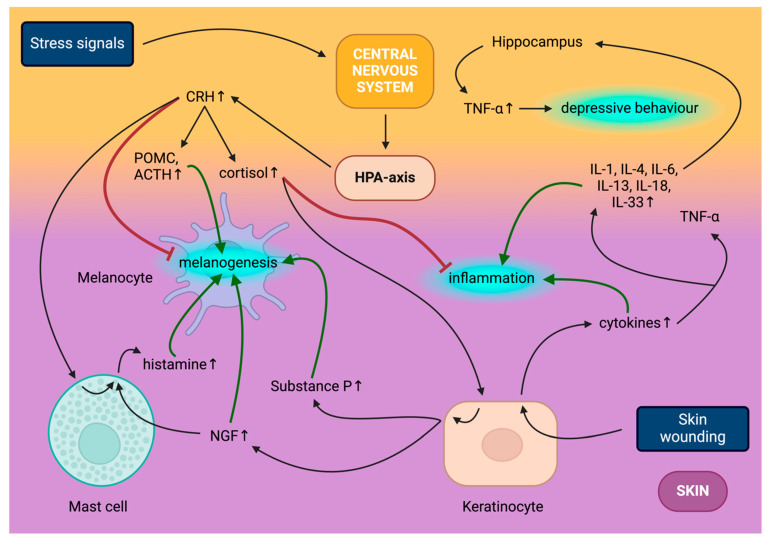
Interplay of central nervous system (CNS) and skin. Stress signals, including psychological stress, employ the hypothalamic–pituitary–adrenal (HPA) axis to produce corticotrophin-releasing hormone (CRH), which induces proopiomelanocortin (POMC) and adrenocorticotropic hormone (ACTH) and increases circulating cortisol production. CRH directly inhibits melanogenesis, meanwhile inducing histamine secretion from mast cells and through POMC and ACTH synthesis indirectly promotes melanogenesis. Cortisol stimulates Substance P and nerve growth factor (NGF) release from keratinocytes, both promoting melanogenesis, while the latter concurrently elevates mast cell histamine secretion. Skin wounding acts on several skin cell types, predominantly keratinocytes, to release cytokines, including tumour necrosis factor alpha (TNF-α), and to release several interleukins (IL), namely IL-1, IL-4, IL-6, IL-13, IL-18 and IL-33 as part of the inflammatory process. Circulating cortisol inhibits multiple skin inflammatory signals and circulating interleukins trigger TNF-α production in the hippocampus, contributing to the onset of depression.

The central HPA axis is triggered in time with the sympathetic nervous system as part of the response to stressors [83]. The main component of the hormonal cascade of the HPA axis is corticotrophin-releasing hormone (CRH); once its production in the hypothalamus is triggered by stress [84], it then goes on to induce the release of pituitary pro-opiomelanocortin (POMC) and POMC-derivatives such as ACTH, α-MSH and β-endorphin (Figure 2) [85]. ACTH secretion elevates adrenal cortisol, corticosterone and glucocorticoid levels as part of homeostasis regulation by binding to melanocortin 2 receptor (MC2R) [84,86]; meanwhile, CRH activates the sympathetic nervous system [87].

The aforementioned glucocorticoids are effector molecules of the HPA axis, normally secreted in a circadian pattern, and regulate the HPA axis via feedback inhibition [88]. Cortisol mediates multiple stress responses by regulating blood pressure homeostasis, the immune system, anti-inflammatory action and the metabolism of protein, adipose and carbohydrates [89]. Cortisol levels oscillate daily under normal circumstances, regulated by the circadian rhythm, peaking in the morning and dropping at midnight in healthy HPA axis subjects [90]. Yet, stress upregulates cortisol levels, and this elevation acts as an immunosuppressant, impacting cytokine and antibody secretion [81].

While acute stress induces a flight-or-fight state, chronic stress, by the same mechanism, has deleterious effects on immune, cardiovascular, metabolic and neural functions [91]. Stress also aggravates numerous neuroinflammatory conditions in the skin, such as psoriasis, atopic dermatitis, acne and erythema [81]. These conditions induce psychological stress, and in turn, neurogenic inflammatory mechanisms amplify, as in a vicious cycle [92].

### 4.2. Peripheral HPA Axis

The peripheral equivalent of the HPA axis in the skin has been identified through the activity of CRH, urocortin, ACTH, α-MSH and β-endorphin, paired with their respective receptors found in skin cells [88,93]. As a response to psychological stress, UVR, cytokines (immune) and skin diseases, keratinocytes, sebocytes and mast cells secrete CRH, which elicits further responses in other skin cell types through multiple CRH receptors (CRH-R1, CRH-R2, belonging to G protein-coupled receptors) [94]. CRH-R1 is produced in both epidermal and dermal compartments [95]. Regarding melanogenesis, CRH signalling triggers the cAMP pathway in melanocytes, producing ACTH and corticosterone, acting similarly to α-MSH [96]. POMC and POMC derivatives bind to melanocortin receptors (e.g., MC1R, MC2R, belonging to the rhodopsin family of transmembrane receptors) [97]. MC1R is expressed in keratinocytes, melanocytes and adipocytes in the skin, and through its mediation, α-MSH triggers anti-inflammatory and pigmentary responses [98,99]. At the same time, ACTH (specific to MC2R) partakes in melanogenesis and regulates the hair cycle’s anagen phase [88]. It has been found that the locally produced inflammatory cytokines secreted during skin wound healing are capable of signalling stress to the brain and result in hippocampal TNF-α production, associated with changes in neuroplasticity, behaviour and cognitive function, factoring into the onset of depression (Figure 2) [100].

## 5. Skin Effects on the CNS—Impact of Hair Colour on Pain

### 5.1. MC1R: Skin–Brain Link

MC1R is predominant in the skin, where, aside from melanogenesis, MC1R also partakes in immune response, DNA repair and cell differentiation and proliferation via both pigment-dependent and -independent pathways and is also expressed in the brain, offering associations between certain pigmentary and neuronal health phenomena [101], such as the effects of α-MSH via the neuronal MC1R counterpart in ischemic stroke [102], traumatic brain and spinal cord injury [103,104], Alzheimer’s disease [105], Parkinson’s disease [106] and neuroinflammatory diseases as a neuroprotective agent. MC1R expressed in immune cells also activates protection against the latter, modulating immune and inflammatory responses [73].

### 5.2. MC1R and Pain

Red hair is most commonly the phenotypical expression of the loss-of-function mutation of the melanocortin-1 receptor (MC1R) gene [107]. Loss-of-function variations in MC1R receptors render MCR1 unresponsive to α-MSH. As a consequence, low MC1R activity will cause low Tyr activity in melanocytes, resulting in melanocytes lacking eumelanin and dominated by pheomelanin instead, resulting in red hair, fair skin and the inability to tan [108,109].

Mutations of MC1R have also been associated with increased sensitivity to noxious thermal stimuli and simultaneous resistance to analgesics such as lidocaine and desflurane [110] hypothetically due to MC1R expression on the surface of brain glial cells and neurones of the ventral periaqueductal grey aside from melanocytes and their involvement in nociception [108]. A more distinct discovery, however, is that homozygous loss-of-function MC1R allele-bearing (MC1R variant) humans and mice show high nociceptive thresholds in line with previous genotype-driven studies [111].

Based on observations made most prominently by dentists in the past decades, non-opioid analgesic requirements, such as those of lidocaine and desflurane, are not usually satisfied by the routine dosage [110,112,113]. Meanwhile, opioid analgesics are associated with enhanced nociceptive responses due to the loss of melanocortin signalling in MC1R deficiency [114,115,116].

### 5.3. Mechanism of Altered Nociception in Red-Haired Background

Our group recently described the mechanism of MC1R-associated altered nociception [116]. It was demonstrated that MC1R regulates POMC expression in melanocytes, and POMC-derived MSH will then modulate central nociception. MC1R loss-of-function implies that α-MSH is rendered non-stimulatory as a ligand, resulting in a low cAMP signalling cascade. Therefore, melanocyte-specific POMC expression is decreased [116,117]. The low cAMP levels hence equate to lowered POMC levels and lower levels of its derivatives, such as ACTH and α-MSH [118]. Conversely, albeit less effectively, POMC and ACTH can induce cAMP production via non-mutant MC1R [109,119,120]. These results collectively suggest a positive autocrine feedback loop in melanocytes, where MC1R signalling stimulates melanocyte-specific POMC and α-MSH secretion, which will further increase its expression by binding to MC1R [116].

**Figure 3:**
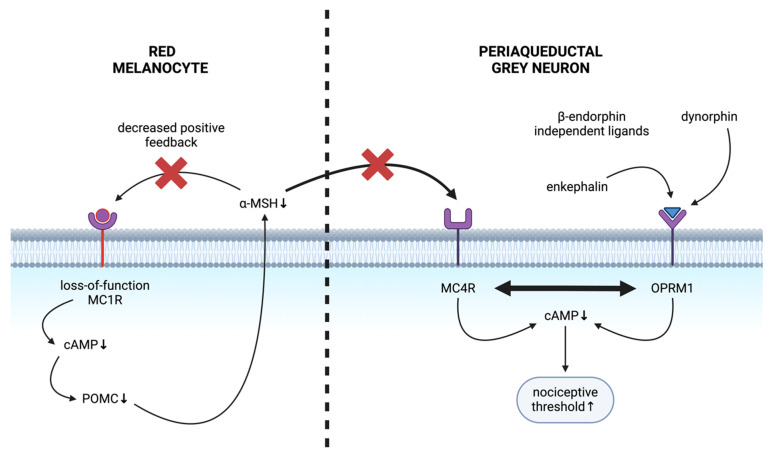
Mechanism of altered nociception in the red-haired background. Loss-of-function melanocortin-1 receptors (MC1Rs) render melanocytes insensitive to alpha-melanocyte stimulating-hormone (α-MSH). The autocrine feedback loop of α-MSH is also compromised, ultimately leading to α-MSH depletion in MC1R deficiency. Decreased peripheral α-MSH leads to decreased central melanocortin-4 receptor (MC4R) signalling, lowering intracellular cAMP in neurons in the periaqueductal grey area, leading to increased nociceptive thresholds. μ-opioid receptor (OPRM1) binds β-endorphin-independent opioid ligands to maintain opioid receptor signalling and to increase nociceptive thresholds. Hence, the antagonistic balance of MC4R and OPRM1 signalling shifts towards an elevated nociceptive threshold in MC1R deficiency.

Mechanistically, the diminished MSH expression in the red-haired genetic background increases nociceptive thresholds that depend on opioid signalling and the absence of melanocortin signalling [116]. When exposed to α-MSH at levels within the normal range for non-mutant MC1R signalling, melanocortin-4 receptor (MC4R) reduces nociceptive thresholds [121]. Given that μ-opioid receptor (OPRM1) inhibition restores the nociceptive threshold in MC1R mutant red-haired mice, similarly to melanocortin agonist treatment (activating MC4R), it was found that the nociceptive threshold is determined by the balance between OPRM1 and MC4R signalling (Figure 3) [116]. The elevation of nociceptive thresholds in MC1R variants is due to the effects of POMC depletion on this balance and the resulting decrease in central MC4R signalling [116]. Multiple central brain regions have been implicated in expressing MC4R and were experimentally found in the periaqueductal grey [122]. They mediate increased nociceptive thresholds in the red-haired genetic background; however, other regions may also contribute to the phenotype [116].

Our group recently identified centrally located MC4R receptors to be required to alter nociception in the MC1R loss-of-function red-haired genetic background [116]. MC4R employs the melanocortin pathways through α-MSH and ACTH binding, among others, in the case of red hair, modulating sensitivity to noxious stimuli [123]. Loss of central MC4R signalling in red-haired mice is due to the loss of peripheral melanocytic production of POMC-driven alpha-MSH. In line with these observations, high nociceptive thresholds were rescued by restoring central melanocortin signalling in red-haired mice [116]. While administering an MC4R antagonist reversed thermal and mechanical hyperalgesia in rats, α-MSH administration resulted in thermal hyperalgesia in naive rats, and the chronic inhibition of MC4R both reversed the opioid effects of morphine and reversed morphine-induced hyperalgesia [124]. Furthermore, the loss of melanocortin agonism via MC4R offered a novel therapeutic intervention through OPRM1 signalling, as it was found that multiple central brain regions co-express OPRM1 and MC4R, two G-protein coupled receptors that seemed to antagonise each other [116].

OPRM1 partakes in endogenous and exogenous opioid response regulation in the central nervous system [125], ultimately inhibiting neurotransmitter release and, thus, the transmission of nervous stimuli upon binding [126]. While β-endorphin, a POMC-derivative and endogenous OPRM1 ligand [127], was not found to affect nociceptive thresholds in MC1R wild-type animals directly, OPRM1 loss-of-function decreased nociceptive thresholds of MC1R mutant animals, suggesting that MC1R modulates nociception in an OPRM1-dependent manner [116]. However, the plasma levels of another derivative of POMC, α-MSH, are also decreased in MC1R variant yellow-furred (equivalent to red-haired) mice. Normally, the melanocortin pathway is capable of antagonising opioid pathways via α-MSH. Therefore, α-MSH depletion in MC1R mutant animals explains the elevated nociceptive thresholds [116,128]. The receptor identified to be responsible for modulating nociception via the melanocortin pathway in the red-haired genetic background is MC4R [116].

## 6. Skin Effects on the CNS—Skin-Derived Endogenous Opioids Driving Addiction

**Figure 4:**
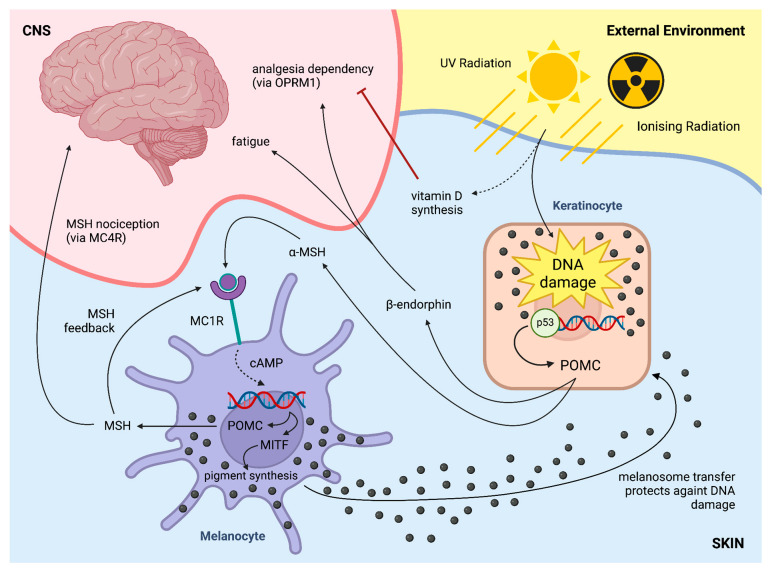
UV-induced melanogenesis and behaviour effects. Ultraviolet (UV) radiation triggers vitamin D synthesis, and, along with ionising radiation, damages DNA in keratinocytes. This induces p53-mediated proopiomelanocortin (POMC) transcription. A derivative of POMC, β-endorphin, is responsible for central fatigue. Melanocortin-1 receptor (MC1R)-mediated pigment synthesis is also triggered by the POMC derivative MSH. Melanocytes produce melanocyte-stimulating hormone (MSH) which triggers further MSH production in an autocrine positive feedback loop. Plasma levels of MSH then modulate nociception via melanocortin-4 receptor (MC4R) in the central nervous system (CNS).

It has been suggested that UV radiation can be addictive [13]. Further mechanistic preclinical studies implicated the role of the pigment pathway in mediating the reinforcing effects of UV radiation (Figure 4): as UV radiation promotes POMC transcription, not only will MSH promote pigment production in melanocytes, but POMC-derived β-endorphin will increase in the serum of animals to promote opioid-like addictive behaviour, including antinociceptive and pro-reinforcing effects [13,129].

Similar opioid-like dependency has been observed in people who visit tanning beds frequently [130]. Currently, there is no evidence to substantiate that the effects of artificial UV exposure or natural UV light on addictive behaviour may differ. However, natural light contains wavelengths that may have additional, known physiologic effects, such as blue light which regulates melatonin production in the circadian rhythm [131]. It is possible that natural UV light may present additional behavioural phenotypes compared to artificial UV light. The UVR-β–endorphin pathway thus promotes addictive behaviour to one of the most ubiquitous carcinogens, UV radiation, although UV-induced pigmentation is an evolutionary, highly conserved pathway across multiple species (including nocturnal animals, e.g., mice and rats); therefore, it is unlikely that the addictive nature of UV radiation may be a purely evolutionary trade-off.

Recently, it has been investigated whether the evolutionary driver of UV-seeking behaviour may be the promotion of vitamin D synthesis [129]. In the context of vitamin D deficiency, maximising its synthesis may carry an evolutionary advantage, especially in younger individuals to prevent rickets [132], despite later negative, possibly lethal consequences like skin cancer, which mainly occurs at post-reproductive ages with no effect on population fitness. Although vitamin D deficiency-associated rickets nowadays does not pose a significant selection pressure to human populations, the association between vitamin D and multiple diseases (including cancer, cardiovascular risk, autoimmunity, etc.) suggests a pivotal role of vitamin D in overall health [133]. This role is also supported by the notion that even in recent human evolution, vitamin D deficiency served as a driver for pigment-related genes [134], as a lighter skin tone allows for increased vitamin D synthesis in higher latitudes [135]. These observations collectively suggest that UV radiation-induced β-endorphin synthesis may have been an evolutionarily beneficial pathway to prevent vitamin D deficiency. However, this pathway comes with a trade-off that is clinically relevant to healthcare systems: vitamin D deficiency increases not only β-endorphin but also opioid addictive behaviour as well [129]. As a trade-off, in patients with low vitamin D levels, exogenously administered opioids may exert heightened effects similarly to increased sensitivity to endogenous endorphin-mediated reward [129]. However, in the case of exogenously administered opioids, the negative feedback from the UV–endorphin paralleled vitamin D synthesis was absent. Therefore, vitamin D-deficient individuals may be at higher risk for developing maladaptive opioid-dependent behaviour. This observation has also been supported by a few independent population-based studies [129,136,137]. Thus, UV radiation-induced endorphin elevation may trigger opioid-related central effects, e.g., reward and dependency, to prevent vitamin D deficiency by promoting maximal vitamin D synthesis through sun-seeking behaviour.

Recently, the effect of keratinocyte-driven POMC and endorphin release to manifest as fatigue has been described [11]. Physiological fatigue triggered by initial DNA damage in keratinocytes may explain the anecdotally reported fatigue after a long day of sun exposure at the beach. However, this pathway may have pathologic consequences: extensive DNA damage of the skin, which can occur after extensive ionising radiation utilised in certain cancer treatments, may lead to fatigue [11]. Although ionising radiation leads to the elevation of multiple cytokines and triggers severe tissue damage, that may all contribute to fatigue behaviour [138,139]; nonetheless, the preclinical animal models suggest an independent role of skin-derived β-endorphin in mediating fatigue after localised ionising radiation [11]. This pathway may also explain the anecdotal origins of the sensation of excessive fatigue after spending a long day at the beach with UV exposure—if UV-triggered endorphin release may trigger similar fatigue-like effects (Figure 4).

## 7. Future Directions

This review summarises the most important interactions between skin pigmentation and CNS signalling, including, but not limited to, neural and hormonal mechanisms. A better understanding of the pathways bridging skin-derived signals to seemingly distant diseases may provide potential novel therapeutic options and suggests an opportunity for the identification of increased risk for certain co-morbidities linking pigmentation to disease.

Novel therapeutic options could include using MC4R agonists and OPRM1 antagonists in red-haired individuals to restore nociception. Furthermore, the recent identification of skin-derived endorphin-dependent pathways leading to fatigue may suggest the use of opioid antagonism in patients where excessive irradiation of the skin is suspected to play a pathological role in mediating fatigue.

Studying brain–skin interactions also highlights the option for screening for potential co-morbidities. An example is Parkinson’s disease, where, due to an elevated melanoma risk, more comprehensive skin checks may be warranted for certain patients. Also, due to the vitamin D-mediated antagonism of skin-derived endorphin effects, patients with low vitamin D levels may be at increased risk for developing opioid use disorder. Therefore, vitamin D supplementation merits consideration for patients undergoing major surgeries to mitigate the risk of developing opioid use disorder due to vitamin D deficiency.

However, it is necessary to note that the neurological and physical symptoms a patient displays are often not part of a causative relationship but correlate due to the morbidity of a shared regulatory intermediate, impacting both pigmentation and the CNS (via MC1R). Collectively, these results highlight the importance of studying skin–brain interactions and suggest multiple novel therapeutic strategies in various areas of disease.

## Figures and Tables

**Table 1:** Paracrine regulators of major signalling pathways in melanogenesis.

Secretory Cell	Paracrine Factor	Corresponding Receptor	Signalling Pathway
melanocyte, keratinocyte	β-endorphin	OPRM1	PKC
	α-MSH (alpha-melanocyte-stimulating hormone)	MC1R	PKA
	β-MSH (beta-melanocyte stimulating hormone)	MC4R	
	BMP6 (bone morphogenetic protein 6)	BMPR1/2	MAPK
	BMP4 (bone morphogenetic protein 4)		
keratinocyte	Glu (glutamate)	mGLUR6	PKC
	EDN1 (endothelin 1)	EDNRB	
	norepinephrine	ADRB2	
keratinocyte, fibroblast	FGF (fibroblast growth factors)	FGFR	MAPK
	HGF (hepatocyte growth factor)	MET	
	SCF (stem cell factor)	KIT	
melanocyte, keratinocyte, fibroblast	CRF (corticotropin-releasing factor)	CRF-1R	PKA, PKC
fibroblast	NRG1 (neuregulin 1)	ErbB	MAPK
	WNT (Wingless-type protein)	Fzd-3	WNT

Based on the review from [8]. Receptor abbreviations: melanocortin-1 receptor (MC1R), melanocortin-4 receptor (MC4R), BMPR1A bone morphogenetic protein receptor type 1 and type 2 (BMPR1/2), metabotropic glutamate receptor 6 (mGLUR6), endothelin receptor type B (EDNRB), β2-adrenergic receptor (ADRB2), fibroblast growth factor receptors (FGFR), MET receptor tyrosine kinase (MET), stem cell factor receptor (KIT), corticotropin-releasing factor type 1 receptor (CRF-1R), erythroblastic leukaemia viral oncogene homologue receptor (ErbB), and frizzled-3 receptor (Fzd-3). This table aims to demonstrate that the signalling pathways involved in pigment synthesis are versatile and include numerous skin cell types. Pathways may also overlap, as seen with corticotropin-releasing factor (CRF), which is secreted by melanocytes, keratinocytes and fibroblasts as well, to trigger downstream production of proopiomelanocortin and its derivatives.
